# Ultrasonic Elastography Features of Phyllodes Tumors of the Breast: A Clinical Research

**DOI:** 10.1371/journal.pone.0085257

**Published:** 2014-01-15

**Authors:** Lu-Jing Li, Hong Zeng, Bing Ou, Bao-Ming Luo, Xiao-Yun Xiao, Wen-Jing Zhong, Xin-Bao Zhao, Zi-Zhuo Zhao, Hai-Yun Yang, Hui Zhi

**Affiliations:** 1 Department of Ultrasound, Sun Yat-sen Memorial Hospital of Sun Yat-sen University, Guangzhou, Guangdong, China; 2 Department of Pathology, Sun Yat-sen Memorial Hospital of Sun Yat-sen University, Guangzhou, Guangdong, China; University of North Carolina School of Medicine, United States of America

## Abstract

The purpose of this study was to analyze the ultrasonic elastography features of phyllodes tumors of the breast comparing with fibroadenomas. A retrospective database was queried for the patients diagnosed as phyllodes tumors and fibroadenomas at Sun Yat-sen Memorial Hospital from January 2008 to August 2012. Three hundred and fifty lesions from 323 consecutive patients were included in the study. All the cases were examined by conventional ultrasonography and ultrasound elastography. Ultrasound elastography was used to calculate strain ratio of the lesions with bilateral breast tissue at the same depth as reference. There were 36 phyllodes tumors (27 benign, 8 borderline, 1 malignant) and 314 fibroadenomas (158 the pericanalicular type, 103 the intracanalicular type, 53 other special types). The strain ratio for phyllodes tumors (3.19±2.33) was significantly higher than for fibroadenomas (1.69±0.88) (p<0.05). The Spearman^.^s correlation coefficient between strain ratio of ultrasound elastography and pathological groups was significant, with a value of 0.17 (p<0.05). Ultrasound elastography could provide additional information to differentiate phyllodes tumors from fibroadenoma in breast.

## Introduction

Phyllodes tumor of breast is a rare breast neoplasm tumors, accounting for 0.3–1.0% of breast tumors [1], and in the majority of cases is benign. Diagnosis of phyllodes tumor is difficult because many characteristics of this neoplasm are also typical for other changes within the breast, especially for fibroadenoma. Fibroadenoma is a frequent, benign, fibroepithelial tumor. The incidence peaking age of fibroadenoma is between the 2^nd^ and 3rd decades [2]. Imaging diagnostics and histopathological examination are obviously very important in the process of establishing a diagnosis. At mammography, phyllodes tumors often present as well-defined, round or lobulated masses [3]–[5], resembling fibroadenomas [6]–[7]. Most of Chinese women have smaller and denser breasts than Western women [8]–[9]. The sensitivity of mammographic detection diminishes in women with radiographically dense breasts [10]–[12]. So sonography is employed by most physicians to diagnose breast lesions in Chinese women. At sonography, phyllodes tumors often present as well-defined, lobulated tumors with mildly hypoechoic internal echoes, smooth margins and had an heterogeneous internal echo pattern. It is also difficult in differentiating phyllodes tumors and fibroadenoma with ultrasound. Core biopsy has been found to be a reliable tool for evaluating masses of the adolescent breast [13], but it requires an experienced pathologist. Core biopsy cannot always differentiate between phyllodes tumours and fibroadenomas. This distinction is important because, unlike fibroadenomas, phyllodes tumors require wide excision with negative margins [14]. Finally, incisional biopsy can confirm a diagnosis [15]. But it is difficult to persuade patients to undergo additional radical surgery after local excision and histological diagnosis of the disease and many patients decline to have this. Therefore, accurate preoperative diagnosis is necessary to avoid subsequent surgery.

Ultrasound elastography is a new technology developed in recent years, with which the histological characteristics of lesions are revealed by evaluating the elastic characteristics of the underlined tissue [16]. The basic principle of elastography is when a controlled compression is applied to a tissue of interest, it results in tissue deformation. Hard tissue will deform to a smaller displacement while a softer tissue will have a larger displacement. With this technique, the image is obtained through gradual manual compression of the tissue with a linear-array transducer that allows real-time display of the elastogram as a color image superimposed on the B-mode image, with different color representing different level of elasticity. For over a decade ultrasound elastography has been drawing increased attention for the study of soft tissues with the clinical perspective of enabling early detection of lesions which have pathological changes in tissues [16]–[17]. Many researches found ultrasound elastography could provide additional help in the diagnosis of breast lesions [18]–[19]. Few reports have described the elasticity of phyllodes tumors in a large series [20]. Therefore, we have reviewed the elasticity of phyllodes tumors and compared it with the elasticity of fibroadenoma.

## Methods

### Patients

A retrospective study with 3274 breast lesions from January 2008 to August 2012 was approved by the Institutional Review Board of Ethic Committee of Sun Yat-sen Memorial Hospital and written, signed informed consent was obtained from enrolled patients. The inclusion criteria were the following: female patients of at least 18 years of age with a solid lesion in breast through examining with conventional ultrasound and ultrasound elastography, and with histological confirmation in all cases.

Our study used data of phyllodes tumors and fibroadenomas which included a trial of 324 consecutive patients with 350 lesions for whom conventional ultrasonography and ultrasound elastography imaging were performed. There were 36 phyllodes tumors and 314 fibroadenomas histologically. The mean age was 38.45 years, with a range of 18∼67 years for 36 phyllodes tumor and a mean age of 33.72 years with a range of 18∼72 years for 314 fibroadenoma. The tumors varied in size from 7.70 to 41.70 mm (median 20.28 mm) for 36 phyllodes tumors and from 4.50 to 50.70 mm (median 14.14 mm) for 314 fibroadenoma.

### Equipment

Both the conventional and the ultrasound elastography studies were performed by a radiologist with 10 years of experience in breast imaging and 5 years of experience in ultrasound elastography. All images were acquired with Hitachi HV-900 with a 5–13 MHz linear transducer (Hitachi Medical, Tokyo, Japan).

### Ultrasound elastography examination

For each patient, a bilateral whole-breast sonography was initially performed. Sonograhic features were recorded in a computer database. Then ultrasound elastography was switched on. For elastography, the same depths, focus positions, and gain settings were used as for conventional images. The ultrasound scanner was equipped with an elastography unit, images were presented in a split-screen mode with the conventional images in the right, and the translucent color-scale elastography images were superimposed on the corresponding ultrasound image on the left. A square region of interest was set for elastography acquisition. The superior margin was set to include more than 5 mm of breast parenchyma adjacent to the targeted lesion. Ultrasound elastography images were obtained by applying repetitive light compression at the skin above the targeted breast lesion. The probe was positioned perpendicular to the skin when applying pressure. For obtaining a stable image, the probe was applied to the breast and focused on the target lesion with a light impression, as the pressure indicator bar showed a value of 2 to 4, and the mean speed of probe movement was once or twice per second during the compressions. Each pixel of the elasticity image was shown as one of the 256 specific colors, representing the extent of strain by using a scale from red, showing areas of greatest strain (i.e.,softest component), to blue, showing no strain (i.e.,hardest component).

Images were saved in a PACS (picture archiving and communications system) as bitmap files on a hard disc.

### Images analysis

All the images were reviewed by the radiologist who did the original examinations and she was blinded to patient identification, clinical history, other imaging results and pathological findings.

The scoring system described by Itoth [18] was used to score the elastograms of lesions from 1 to 5. An elasticity score of 1 indicates strain in the entire hypoechoic lesion (i.e., the entire lesion was evenly shaded in green). An elasticity score of 2 indicates strain in most of the hypoechoic lesions, but with some areas of no strain (i.e., hypoechoic lesions with a mosaic pattern of green and blue). An elasticity score of 3 indicates strain at the periphery of the hypoechoic lesion, sparing the center of he lesion (i.e., the peripheral part of the lesion was green, and the central part was blue). An elasticity score of 4 indicates no strain in the entire hypoechoic lesion (i.e., the entire lesion was blue, but its surrounding area was not included). An elasticity score of 5 indicates no strain in the entire hypoechoic lesion or the surrounding area (i.e., both the entire hypoechoic lesion and its surrounding area were blue).

Calculation of the strain ratio was based on the comparison of the average strain measured in the lesion with the adjacent breast tissue of the same depth [19]. Using proprietary software on the ultrasound machine, the average strain of the lesion was determined by selecting a representative region of interest from lesion and was expressed as A. A corresponding region of interest of adjacent breast tissue of the same depth was then selected, and the average strain was expressed as B. The resultant strain ratio was calculated according to the equation strain ratio = B/A, which reflected the property of stiffness of the lesion.

### Pathological diagnosis

The histology of all the nodules was established with US-guided 14-gauge automated gun core biopsy within 48 hours of US examinations or excision biopsy, on the basis of imaging findings or physician^,^s decision. Only core biopsy results with definitive diagnosis were accepted, those that were inadequate or that had atypical features underwent excision, including 15 fibroadenomas and 8 phyllodes tumors with equivocal core biopsies. When the diagnosis of phyllodes tumors were made by core biopsy or excision, all of them underwent wide excision. Some women whose lesions were diagnosed as fibroadenomas by core biopsy opted for conservative management. They were reviewed every 3 months with physical examination and ultrasound. Imaging follow-ups were performed on 258 (86.33%) of 299 fibroadenomas; median follow-up duration was eleven months (range 7∼18 months) and lesion stability was confirmed in all.

Phyllodes tumors were further subdivided into benign, borderline, and malignant lesions. Fibroadenomas were also divided according to histological presentation into three subgroups: the intracanalicular type (I), the pericanalicular type (II) and other special type. Special varieties of fibroadenoma include fibroadenoma with lactating adenoma, juvenile fibroadenoma, and tubular adenoma [21]. All samples obtained were sent for histological study, and were analyzed by specialized breast pathologists with at least 15 yeas of experience.

### Statistic analysis

The characteristics of elastograms of phyllodes tumors and fibroadenoma were summarized. The scores and strain ratio which occur within each of the different types of pathology of phyllodes tumors and fibroadenomas were recorded. Differences among strain ratio were assessed with the Student^,^s t-test. The best cutoff point was obtained by comparing Youden 's index (sensitivity+specificity−1) determined with receiver-operating characteristic (ROC) curve analysis. And we grouped the two types into four different groups according to the amount of stroma from the least to the most, which were group 1 (fibroadenoma in type II), group 2 (fibroadenoma in type I), group 3 (benign phyllodes tumors) and group 4 (borderline and malignant phyllodes tumors). Spearman analysis was used to analyze the correlation of pathological characters with ultrasound elastography scores. Spearman^,^s correlation coefficient was calculated between groups and amount of stroma. Two-tailed P values of less than 0.05 was considered to indicate a statistically significant difference. Statistical analysis were performed by using the Statistical Package for the Social Sciences (SPSS version 11.5 for Windows, Inc., Chicago, IL, USA).

## Results

350 masses were included in our study. There were 36 phyllodes tumors (27 benign, 8 borderline, 1 malignant) and 314 fibroadenomas (158 in type I, 103 in type II, 53 in other special type). Distribution of elasticity score for phyllodes tumors and fibroadenomas were summarized in Table 1.

**Table 1:** Distribution of elasticity score for phyllodes tumors and fibroadenomas.

Elasticity score	phyllodes tumors	fibroadenomas
1	15	179
2	10	87
3	2	31
4	8	16
5	1	1
total	36	314

### Pathological characters and ultrasound elastography scores of phyllodes tumors and fibroademomas

**Figure 1:**
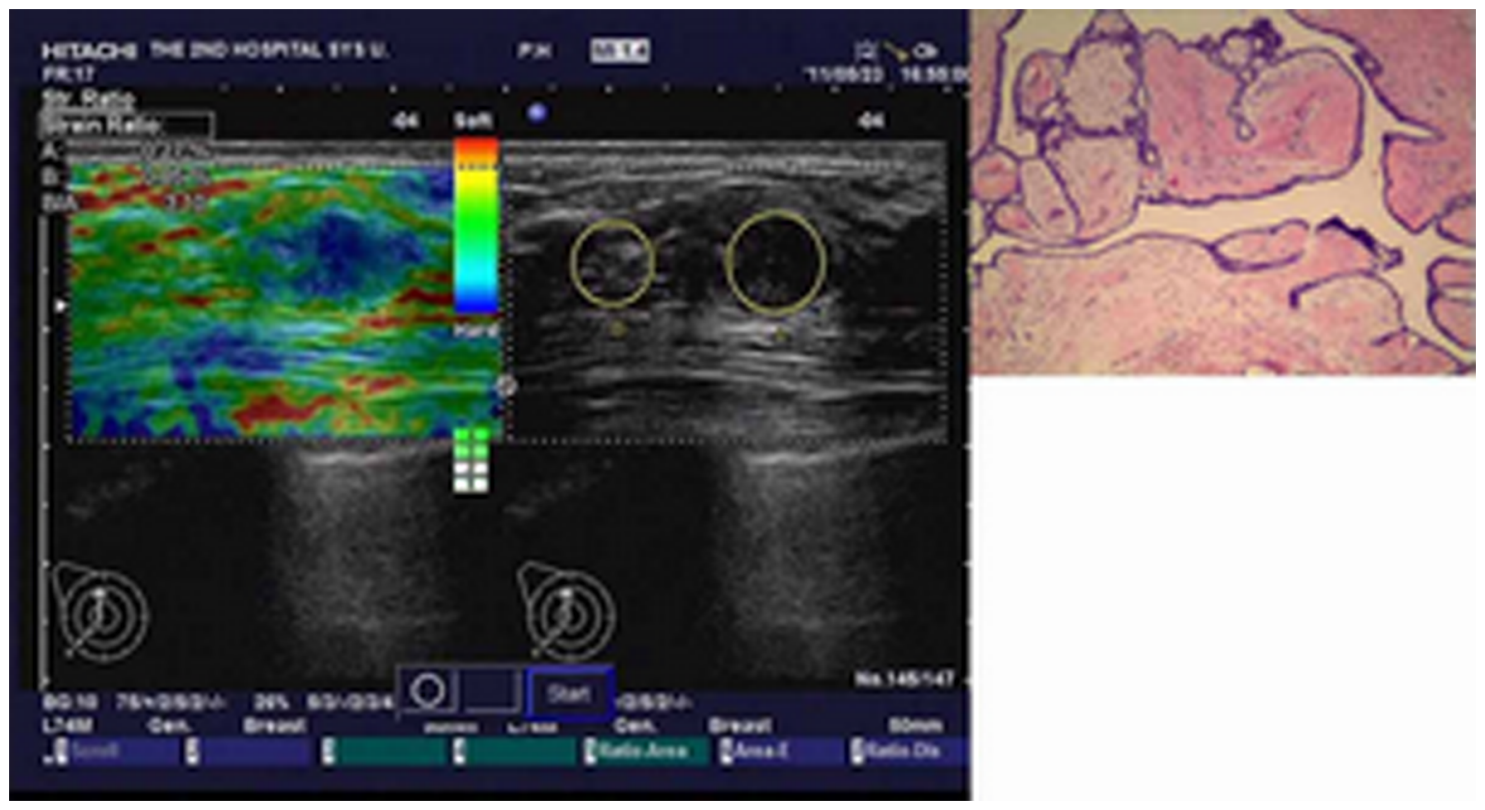
Image of a benign phyllodes tumor in a 42-year-old woman. Left: On elasticity image, the strain ratio was 3.10. Middle: Conventional B-mode image is shown. Right: Pathologic section of lesion is shown.

**Figure 2:**
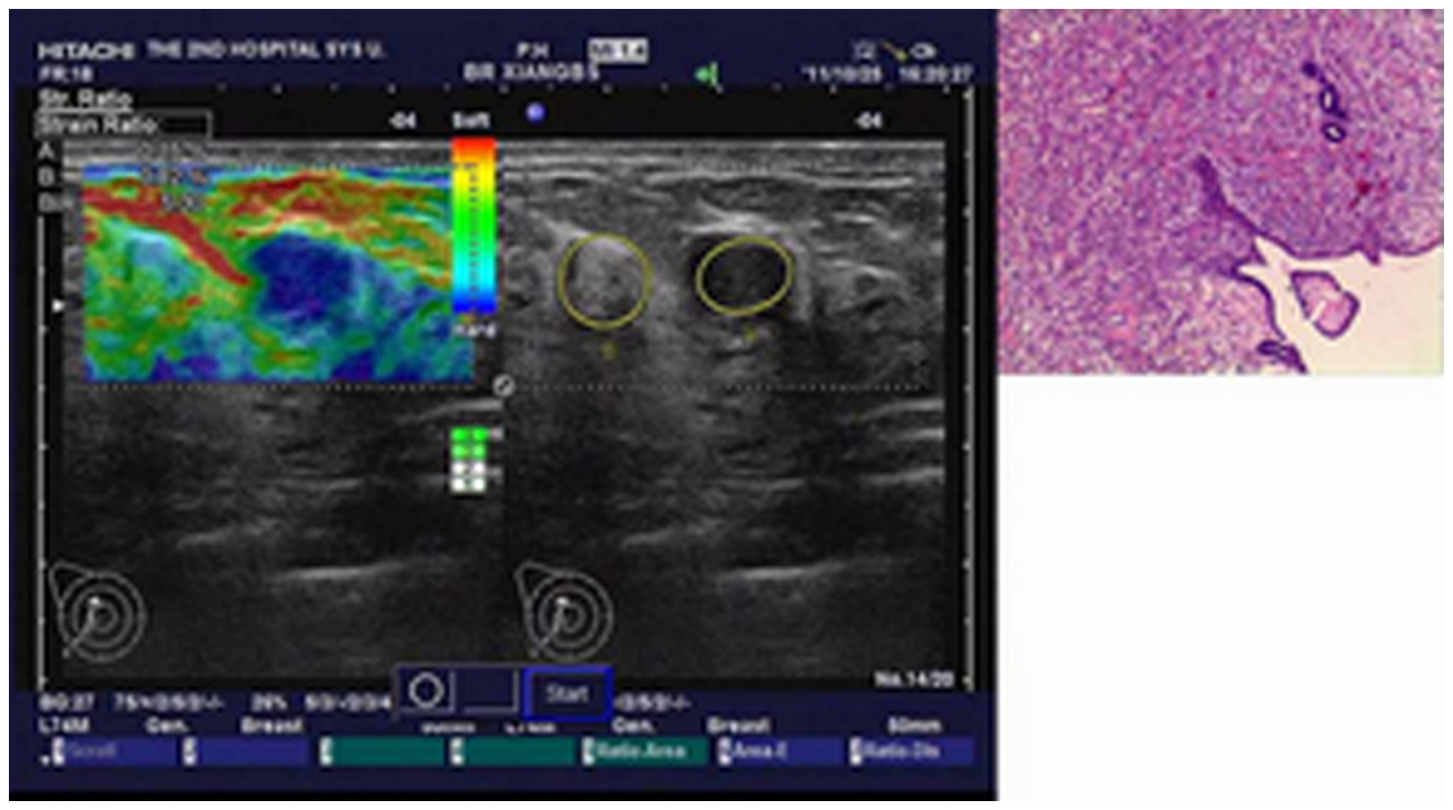
Image of a borderline phyllodes tumor in a 55-year-old woman. Left: On elasticity image, the strain ratio was 5.30. Middle: Conventional B-mode image is shown. Right: Pathologic section of lesion is shown.

When the findings of all 36 phyllodes tumors were analyzed together, 15 (41.67%) were scored as 1, 10 (27.78%) were scored as 2 (Fig. 1), 2 (5.56%) were scored as 3 (Fig. 2), 8 (22.22%) were scored as 4 and 1(2.78%) was scored as 5. Distribution of elasticity score for benign, borderline and malignant phyllodes tumors were summarized in Table 2.

**Table 2:** Distribution of elasticity score for benign, borderline and malignant phyllodes tumors.

Elasticity score	Benign	Borderline	Malignant	Total
1	14	1	0	15
2	8	2	0	10
3	1	1	0	2
4	3	4	1	8
5	1	0	0	1
total	27	8	1	36

**Figure 3:**
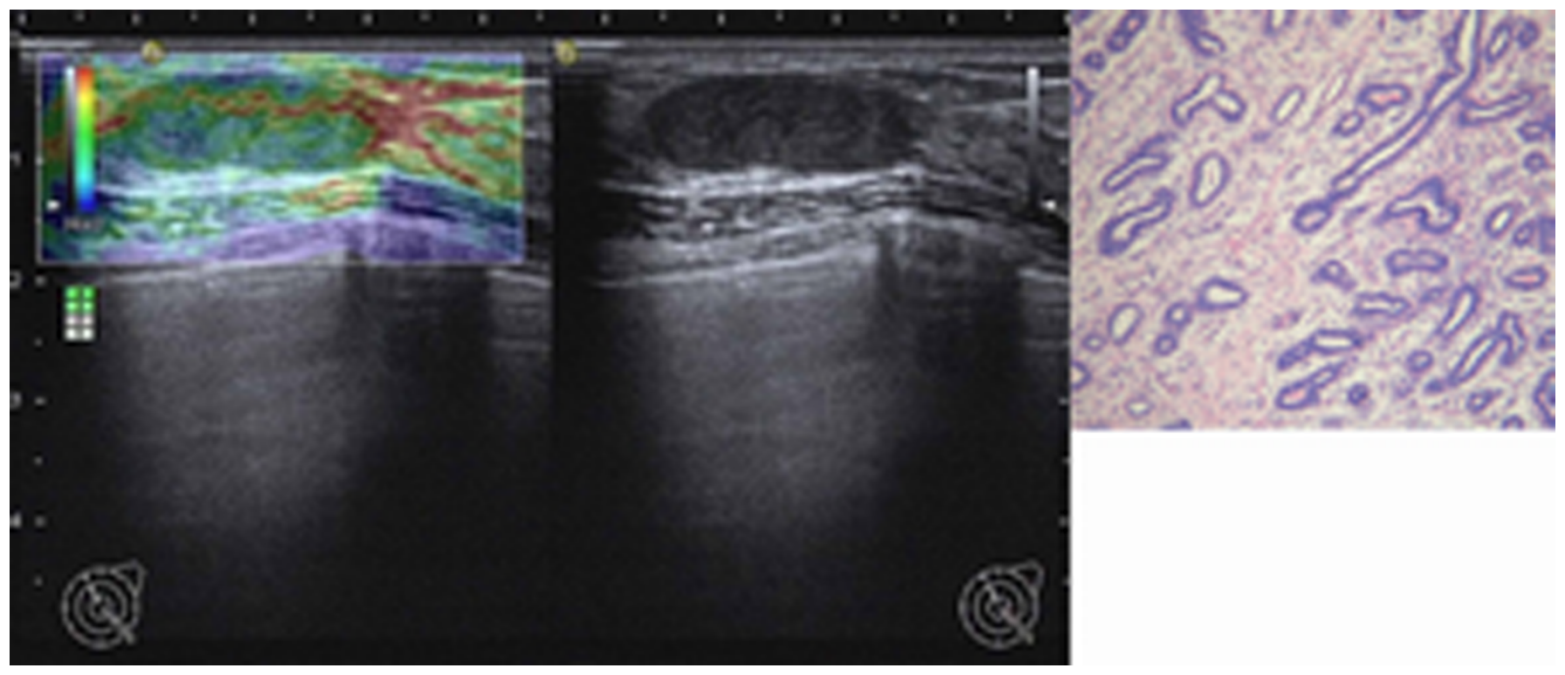
Image of a fibroadenoma in type II in a 29-year-old woman. Left: On elasticity image, the entire lesion was evenly shaded in green (score 1). Middle: Conventional B-mode image is shown. Right: Pathologic section of lesion is shown.

**Figure 4:**
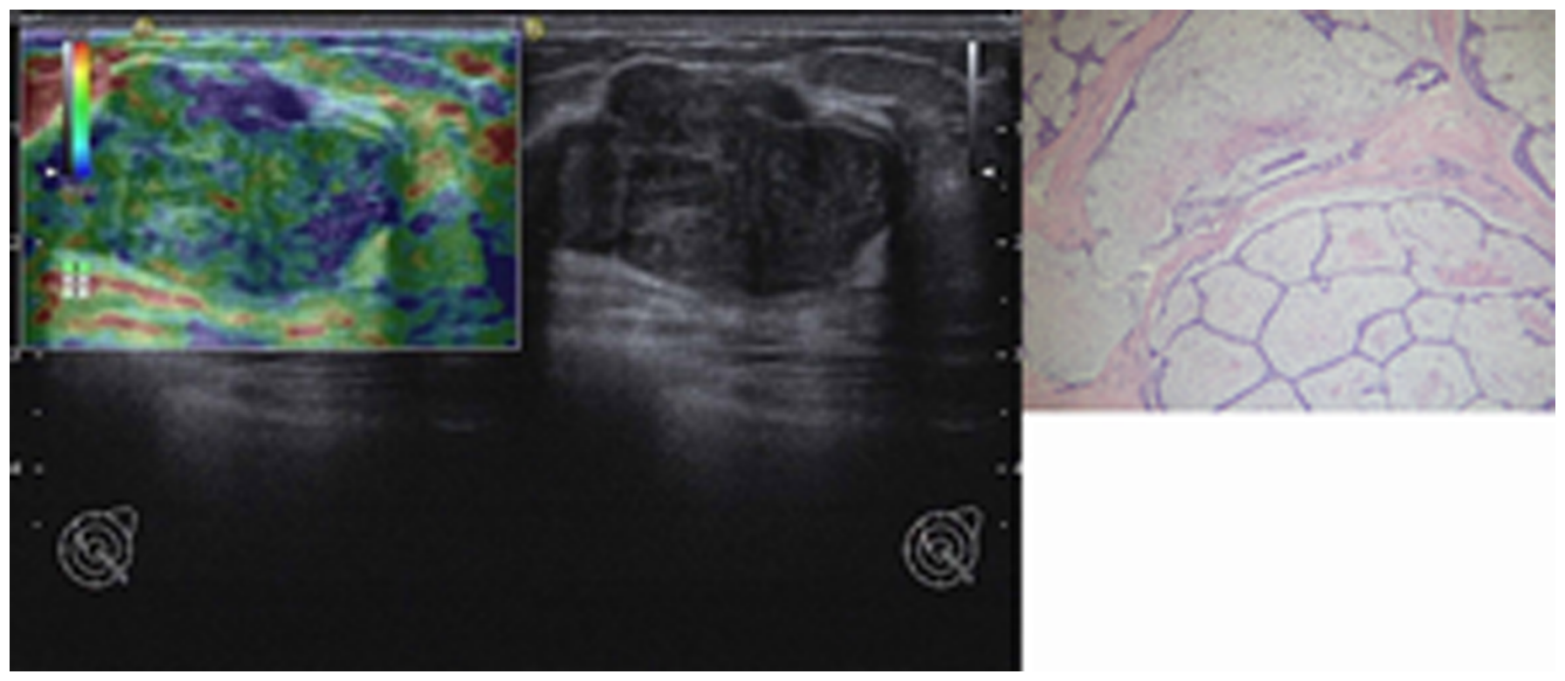
Image of a fibroadenoma in type I in a 25-year-old woman. Left: On elasticity image, most part of the lesion was green, but with some blue ultrasound elastography areas (score 2). Middle: Conventional B-mode image is shown. Right: Pathologic section of lesion is shown.

When the findings of all 314 fibroadenoma were analyzed together, 179 (57.01%) were scored as 1 (Fig. 3), 87 (27.71%) were scored as 2 (Fig. 4) and 31 (9.87%) were scored as 3. 16 (5.10%) had an elasticity score of 4 and 1 (0.32%) had an elasticity score as 5. Distribution of elasticity score for type I, type II and other special type fibroadenomas were listed in Table 3.

**Table 3:** Distribution of elasticity score for type I, type II and other special types of fibroadenomas.

Elasticity score	type I	type II	other type	Total
1	82	64	33	179
2	43	26	18	87
3	20	9	2	31
4	12	4	0	16
5	1	0	0	1
total	158	103	53	314

### Differences of the strain ratio of phyllodes tumors and fibroademomas

The mean strain ratio of 36 phyllodes tumors was 3.19±2.33, while 1.69±0.88 for the 314 fibroadenomas. There were significant differences between the strain ratios of phyllodes tumors and fibroadenomas (P<0.05). Conventionally, a cutoff point is defined as “best” if it attains the maximum of value of Youden 's index. Thus, the best cutoff point in this research was 2.59. With this cutoff point, the sensitivity, specificity, accuracy, positive predictive value and negative predictive value of ultrasound elastography in differentiation phyllodes tumors from fibroadenomas were 52.82%, 89.56%, 82.35%, 29.78% and 94.19%, respectively.

The mean strain ratio for benign phyllodes tumors was 2.94±2.35, while 3.91±2.21 for the borderline and malignant lesions. The strain ratio for benign lesions was significantly lower than for borderline and malignant.

When type I and type II of fibroadenomas were assessed, the following results were obtained: strain ratio of type I was 1.74±1.05 and 1.58±0.58 for type II. There was significant difference between the strain ratio of the two types of fibroadenomas (p<0.001).

### Relationship between strain ratio and histologic parameters of phyllodes tumors and fibroadenomas

There were four groups of all the lesions according to the amount of stroma from the least to the most, they were group 1 (fibroadenoma in type II), group 2 (fibroadenoma in type I), group 3 (benign phyllodes tumors) and group 4 (borderline and malignant phyllodes tumors). The Spearman^.^s correlation coefficient between strain ratio and pathological groups was highly significant, with a value of 0.17 (p = 0.004).

An illustration of a benign phyllodes tumor is shown in Figure 1. An illustration of a borderline phyllodes tumor is shown in Figure 2. An illustration of a fibroadenoma in type I is shown in Figure 3. An illustration of a fibroadenoma in type II is shown in Figure 4.

## Discussion

In this study, we mainly observed the elastography presentation of phyllodes tumors. Phyllodes tumors have both epithelial and stromal components, and phyllodes tumors have similar histological characteristics as fibroadenomas. In addition, imaging findings of phyllodes tumors have been reported insufficiently and the differentiation between fibroadenomas and phyllodes tumors remains difficult [22]–[24]. Overall, we found that there was a variation according to the strain ratio of ultrasound elastography, with a mean of 3.19 for phyllodes tumors and 1.69 for fibroadenoma. Our result demonstrated that phyllodes tumors were firmer than fibroadenomas. It is in agreement with histological findings. It has been reported that there are four histological features that have been found to be useful in the differentiation of phyllodes tumors and fibroadenomas on needle core biopsy of the breast, stromal cellularity increased in at least 50% compared with typical fibroadenoma, stromal overgrowth (at least 10 fields with no epithelium), fragmentation and adipose tissue within stroma [25]. Fibroadenomas usually arise in the terminal ductal lobular unit of the breast gland. The clinical manifestation of phyllodes tumors is most often a firm or hard round tumor. Phyllodes tumors develop from the periductal stroma and contain sparse lobular elements. In comparison with fibroadenomas, phyllodes tumors are characterized by expansion and increased cellularity of the stroma. Therefore, phyllodes tumors are characterized with more abundant and cellular stroma than fibroadenomas. So phyllodes tumors tend to be harder than fibroadenomas according to histological characters. There are several methods of determining the elasticity of a lesion, including the ultrasound-based strain ratio assessment method. It can compare the tissue strain of two interesting regions by analyzing the different color patterns to obtain the strain index (ie, the breast tissue/tumor strain ratio). In our previous clinical study, we found that using the strain ratio assessment method, ultrasound elastography had better diagnostic performance than with the five-point scoring method [26]. During the evaluation of this method, we found that 2.59 was the best cutoff point. However, with this best cutoff point of 2.59, the sensitivity of ultrasound elastography was only 52.82%. We thought it was because that.there were still 15 (41.67%) lesions of phyllodes tumors scored as 1 in our results, including 14 benign and one borderline phyllodes tumors. The reason maybe that benign phyllodes tumors are softer in comparison with borderline and malignant ones and close to fibroadenomas. These lesions may be misdiagnosed as fibroadenomas if only relied on ultrasound elastography results. It is well known that ultrasound elastography is a modality to provide additional information to conventional sonography and be used just as an auxiliary tool in assessing the masses in clinical practice. Further study should be focused on how to combine the results of conventional sonography with ultrasound elastography together.

Although Hongna Tan et al. [27] found several sonographic findings such as irregular shape could be used to help preoperatively determine the histologic grade of phyllodes tumors, most literatures [28]–[29] reported that sonography could not be used to differentiate benign from malignant phyllodes tumors. Our results showed that the strain ratio of ultrasound elastography for benign phyllodes tumors lesions was lower than for borderline and malignant phyllodes tumors. The reason might be that low-grade malignant or borderline lesions and malignant phyllodes tumors show a marked degree of hypercellular stromal overgrowth. The limitation was the small number of malignant lesions of phyllodes tumors in our study, which was due to the rarity of this type of lesion. Only one malignant lesion was found in 36 (2.78%) phyllodes tumors. Further studies that include a larger number of malignant cases are needed.

Our results also found that fibroadenoma in type I appeared firmer at elastography. The reason also correlates with the histological features. Fibroadenomas usually arise in the terminal ductal lobular unit of the breast gland and can be divided into two groups based on the relationship between the breast stroma and the epithelial structures. Pericanalicular fibroadenomas consist of intact round/oval gland spaces and arrange in single or multiple cell layers and concentric arrangement of the adjacent stroma. While intracanalicular fibroadenomas, in which the stroma undergoes more active proliferation, cause compression of the gland tissue and reduction of the lumen. Intracanalicular fibroadenomas with high levels of stromal fibrosis tend to be harder. In this study we only analyzed two types of fibroadenoma because the stroma of other special types has no specific characteristics.

Meanwhile our results show that the elasticity of the four groups were related to the different amount of stroma. We chose these four groups because of their pathological characters. The stroma in type I fibroadenoma undergoes more active proliferation than type II. In comparison with fibroadenomas, phyllodes tumors are characterized by expansion and increased cellularity of the stroma. While low-grade malignant or borderline phyllodes tumors show a marked degree of stromal overgrowth than benign ones. So four groups were divided according to the amount of stroma from the least to the most. The reason for the poor correlation coefficient value might be that ultrasound elastography is lack of enough sensitivity to reflect the amount of stroma sharply.

## Conclusions

In summary, we primarily described ultrasound elastography and pathologic features of 350 lesions. We concluded that ultrasound elastograph could give some help in the differentiation of phyllodes tumors from fibroadenoma in breast. Further study of how to combine sonography and ultrasound elastography results together to differentiate phyllodes tumors and fibroadenoma is required in order to devise management recommendations.
